# *Notes from the Field:* Transmission of Pan-Resistant and Echinocandin-Resistant *Candida auris* in Health Care Facilities ― Texas and the District of Columbia, January–April 2021

**DOI:** 10.15585/mmwr.mm7029a2

**Published:** 2021-07-23

**Authors:** Meghan Lyman, Kaitlin Forsberg, Jacqueline Reuben, Thi Dang, Rebecca Free, Emma E. Seagle, D. Joseph Sexton, Elizabeth Soda, Heather Jones, Daryl Hawkins, Adonna Anderson, Julie Bassett, Shawn R. Lockhart, Enyinnaya Merengwa, Preetha Iyengar, Brendan R. Jackson, Tom Chiller

**Affiliations:** ^1^Division of Foodborne, Waterborne, and Environmental Diseases, National Center for Emerging and Zoonotic Infectious Diseases, CDC; ^2^Center for Policy Planning and Evaluation, DC Health, Washington, DC; ^3^Texas Department of State Health Services; ^4^Division of Healthcare Quality Promotion, National Center for Emerging and Zoonotic Infectious Diseases, CDC.

*Candida auris* is an emerging, often multidrug-resistant yeast that is highly transmissible, resulting in health care–associated outbreaks, especially in long-term care facilities. Skin colonization with *C. auris* allows spread and leads to invasive infections, including bloodstream infections, in 5%–10% of colonized patients (*1*). Three major classes of antifungal medications exist for treating invasive infections: azoles (e.g., fluconazole), polyenes (e.g., amphotericin B), and echinocandins. Approximately 85% of *C. auris* isolates in the United States are resistant to azoles, 33% to amphotericin B, and 1% to echinocandins (*2*), based on tentative susceptibility breakpoints.* Echinocandins are thus critical for treatment of *C. auris* infections and are recommended as first-line therapy for most invasive *Candida* infections (*3*). Echinocandin resistance is a concerning clinical and public health threat, particularly when coupled with resistance to azole and amphotericin B (pan-resistance).

Pan-resistant *C. auris* isolates have been reported previously, although rarely, from the United States (*4*) and other countries (*5*). Three pan-resistant *C. auris* cases reported in New York developed resistance following echinocandin treatment and lacked epidemiologic links or common health care (*4*), suggesting that resistance resulted from antifungal pressure rather than via person-to-person transmission. Since January 2021, however, the Antibiotic Resistance Laboratory Network has detected independent clusters of pan-resistant or echinocandin-resistant cases in Texas and the District of Columbia (DC). Each cluster involved common health care encounters and no known previous echinocandin exposure, suggesting transmission of pan- and echinocandin-resistant strains for the first time in the United States.

Among 101 clinical and screening cases of *C. auris*^†^ in DC during January–April 2021, three had an isolate that was pan-resistant. All resistant isolates were identified through skin colonization screening at one long-term care facility for severely ill patients, including those requiring mechanical ventilation.

Among 22 clinical and screening cases of *C. auris* in Texas during the same period, two were pan-resistant and five were resistant to both echinocandins and fluconazole. These seven cases were identified in patients who were cared for at two facilities that share patients in the same city; two patients were at a long-term acute care hospital, three at a short-term acute care hospital, and two at both facilities. Among these cases, four were identified through colonization screening and three through clinical isolates (two blood isolates and one wound isolate).

No known epidemiologic links were identified between the Texas and DC clusters. No patients with pan- or echinocandin-resistant isolates in either cluster had received echinocandins before *C. auris* specimen collection. Thirty-day mortality in both outbreaks combined was 30%, but the relative contribution of *C. auris* was unclear.

These two simultaneous, independent clusters of pan- or echinocandin-resistant *C. auris* cases in patients with overlapping inpatient health care exposures and without previous echinocandin use provide the first evidence suggesting that pan- or echinocandin-resistant *C. auris* strains might have been transmitted in U.S. health care settings. Surveillance, public health reporting, and infection control measures are critical to containing further spread. Clinicians should consider early antifungal susceptibility testing in patients with *C. auris* infection, especially in those with treatment failure. Data are lacking about the most appropriate therapy for pan-resistant infections. Combination and investigational antifungal treatments can be considered, but evidence in clinical settings is limited (*6*). More information is needed to evaluate patient outcomes and identify proper treatment for *C. auris* cases with pan-resistance or echinocandin resistance.

## Footnotes

*
https://www.cdc.gov/fungal/candida-auris/c-auris-antifungal.html

†
https://ndc.services.cdc.gov/conditions/candida-auris/
